# Pinpointing the Earliest Defects in Age-Related Macular Degeneration

**DOI:** 10.1371/journal.pmed.0030038

**Published:** 2005-11-29

In the developed world, age-related macular degeneration (AMD) is the most common cause of blindness in later life—in the United States, for example, it affects around 15 million people. Early signs of AMD in the retina are pigmentation and soft drusen deposits of protein, fat, and cellular debris. Advanced AMD was previously classified into wet and dry types; dry AMD, now known as geographic atrophy (GA) or atrophic AMD, occurs as the light-sensing cells (photoreceptors) in the macula break down. Wet AMD, now known as neovascular or exudative AMD, is caused when abnormal, fragile blood vessels grow under the macula, underneath the retina. These blood vessels often leak blood, lipid, and fluid, which lift the macula.

Late (sight-threatening) AMD is found in about 2% of all people over 50 years of age, and the incidence of the disease rises with age, occurring in 0.7%–1.4% of people aged 65–75 years, and in 11%–19% of people over 85 years of age. The neovascular form can rapidly lead to severe blindness, whereas the atrophic form progresses more slowly. Although age is the main risk factor for AMD, hypertension, smoking, and a family history of AMD also increase risk of developing the disease.

Previous genetic linkage studies have suggested that a locus on the long arm of Chromosome 1 was involved in AMD's pathogenesis. Further studies, then, refined these analyses and showed that a variant in one gene, Complement Factor H *(CFH)*, was present more frequently in people with advanced AMD than in normal controls. A paper in *PLoS Medicine* now takes this genetic analysis further, asking whether this same variant is also associated with early AMD.

The investigators, from Iceland and the US, looked at two cohorts of patients with advanced and early AMD, and compared them with controls. They confirmed previous work on the association of the *CFH* variant with advanced AMD, but furthermore showed that the same variant was associated with soft drusen and also equally with both forms of advanced AMD. The implications of these findings are that *CFH* would seem to have a role early in the development of AMD, and that other genes or environmental factors are likely to determine which patients will progress to late AMD, and if so, which type of AMD. This role would fit in with what is known about CFH. CFH is a serum glycoprotein that controls the function of the alternative complement pathway and acts as a cofactor with factor I (C3b inactivator). Family syndromes have been described in which deficiency of CFH leads to spontaneous activation of the alternative pathway. The variation associated with AMD causes a milder phenotype, but may attenuate the complement inhibitory function of CFH, making complement attack of retinal pigmented epithelial and choroidal cells via the alternative pathway more likely. [Fig pmed-0030038-g001]

**Figure pmed-0030038-g001:**
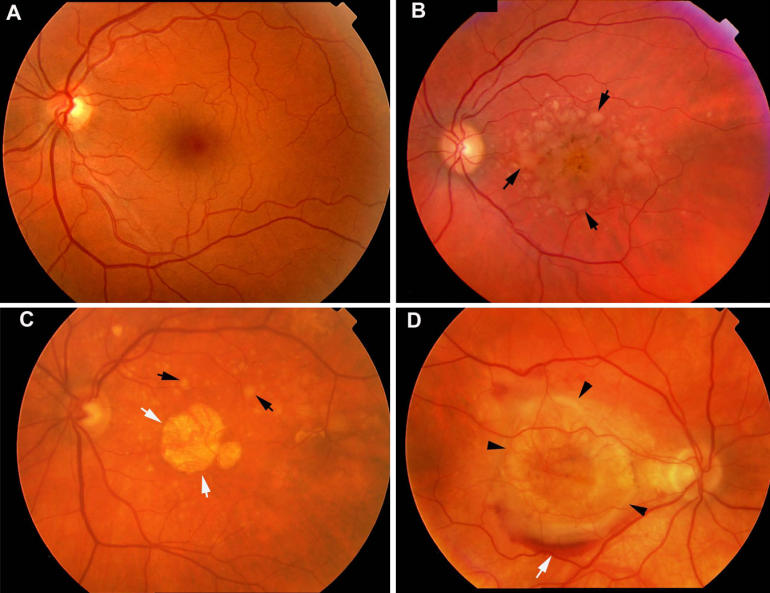
Fundus images of A, normal macula; B, macula with confluent soft drusen C, macula with dry AMD D, macula with wet AMD

Ultimately, work such as this that dissects out genetic risk factors for diseases are of most value when they suggest, as here, a pathway for targeting, in a disease with few treatment options once the disease is established.

